# Supplementary material for: Experimentally Validated Reconstruction and Analysis of a Genome-Scale Metabolic Model of an Anaerobic Neocallimastigomycota Fungus
Source: mSystems. 2021 Feb 16;6(1):e00002-21. doi: 10.1128/mSystems.00002-21 (PMC8561657; doi:10.1128/mSystems.00002-21)
Supplement: TABLE S1 [file msystems.00002-21-st001.docx]

| Fungus | Genome size (Mbps) [# contigs] | Number of predicted genes [% annotated] | Number of genes with EC annotations [# unique ECs] | Number of genes with CAZyme annotations |
| --- | --- | --- | --- | --- |
| *P. finnis* | 56.46 [232] | 10992 [66.8] | 2925 [464] | 573 |
| *N. californiae* | 193.03 [1819] | 20219 [62.4] | 4774 [491] | 1189 |
| *A. robustus* | 71.69 [1035] | 12832 [65.9] | 3343 [479] | 698 |
| ***N. lanati*** | **200.97 [970]** | **27677 [52.3]** | **2761 [490]** | **1788** |
| *P. ruminantium* | 100.95 [32574] | 18936 [54.9] | 3457 [359] | 886 |
| *P. sp. E2* | 71.02 [17217] | 14648 [55.8] | 2585 [408] | 1003 |
| *S. cerevisiae* | 12.07 [16] | 6575 [66.4] | 1662 [573] | 143 |
