# Supplementary figures and images for: Experimentally Validated Reconstruction and Analysis of a Genome-Scale Metabolic Model of an Anaerobic Neocallimastigomycota Fungus
Source: mSystems. 2021 Feb 16;6(1):e00002-21. doi: 10.1128/mSystems.00002-21 (PMC8561657; doi:10.1128/mSystems.00002-21)

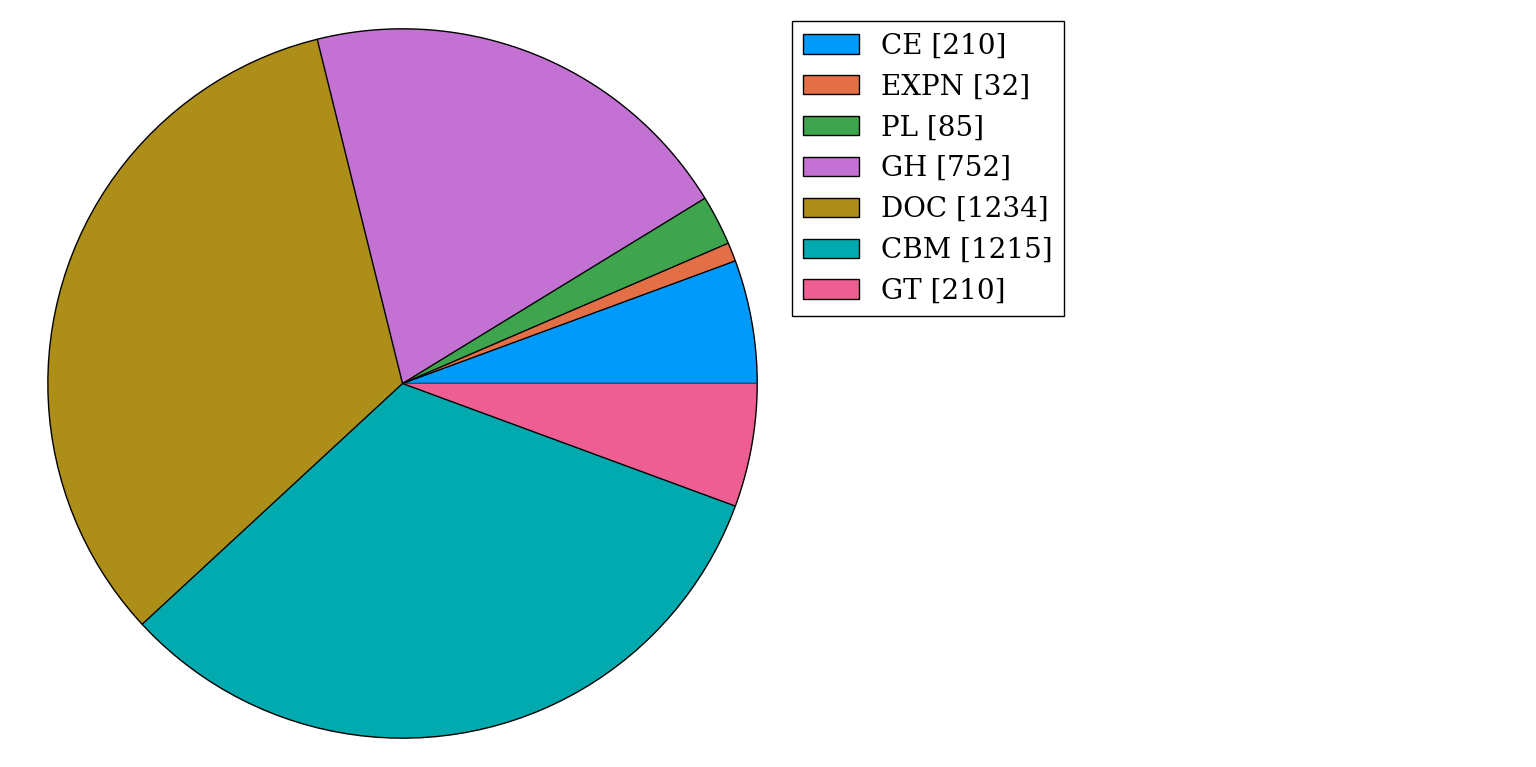

Supplement: FIG S1 [file msystems.00002-21-sf001.tif]

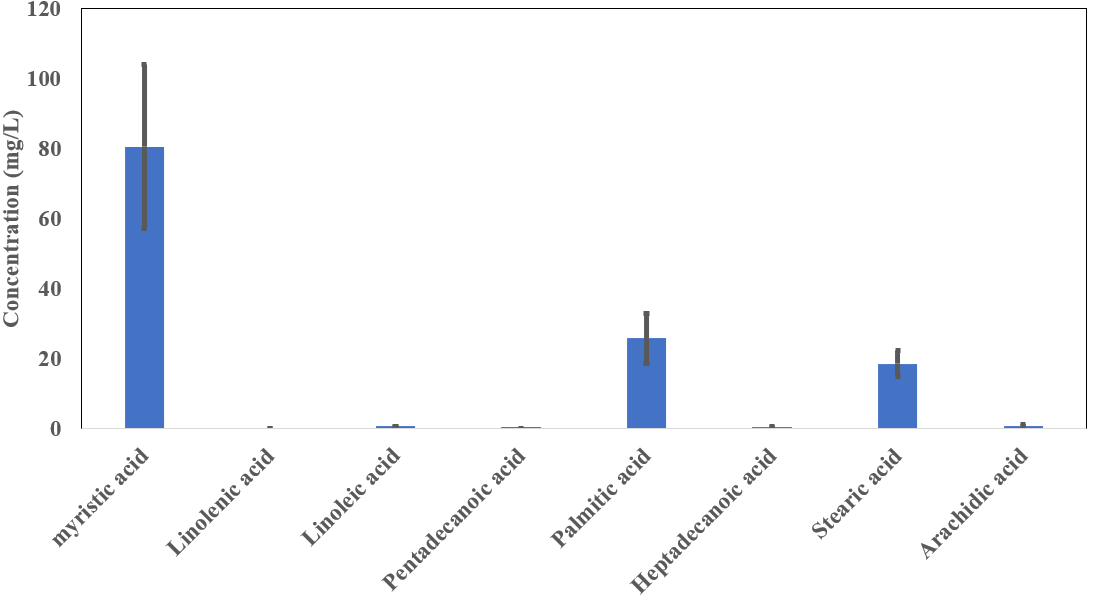

Supplement: FIG S2 [file msystems.00002-21-sf002.tif]

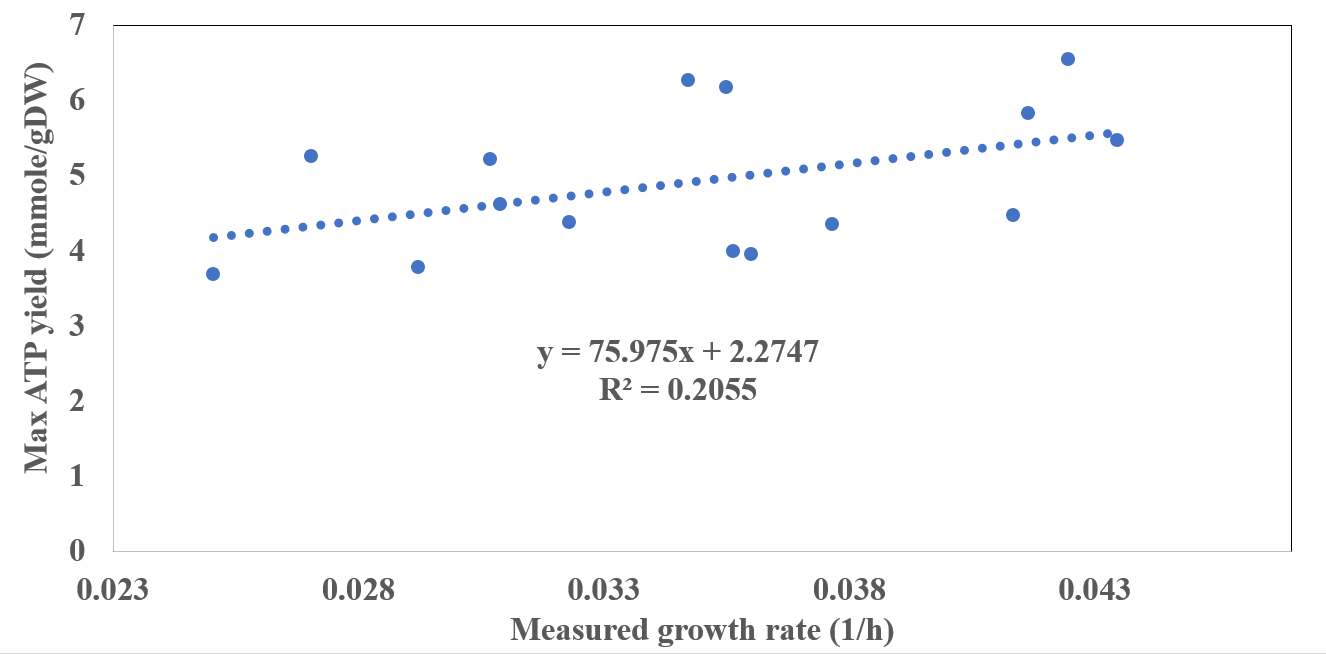

Supplement: FIG S3 [file msystems.00002-21-sf003.tif]

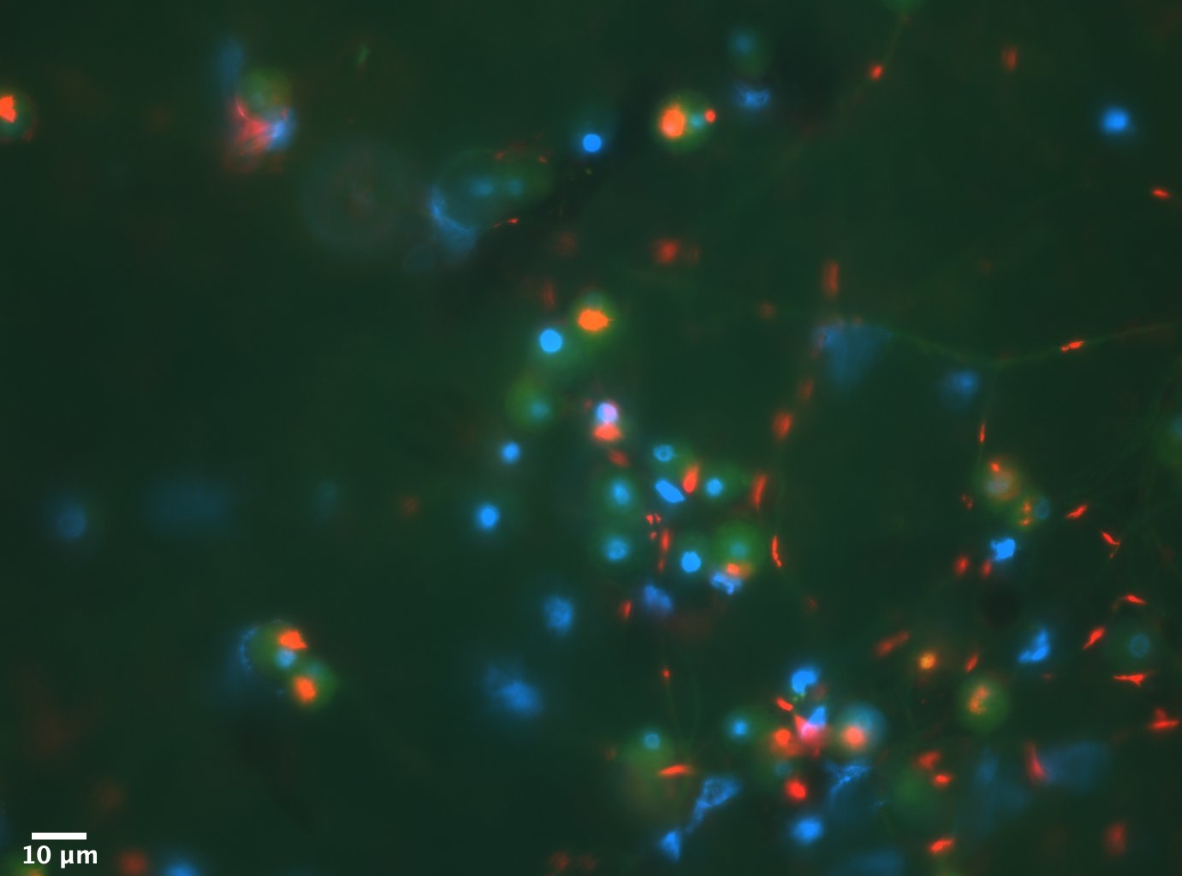

Supplement: FIG S4 [file msystems.00002-21-sf004.tif]

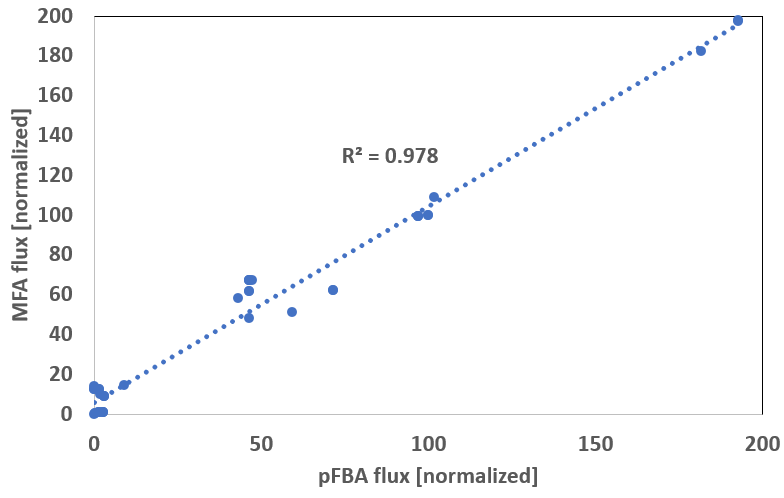

Supplement: FIG S5 [file msystems.00002-21-sf005.tif]

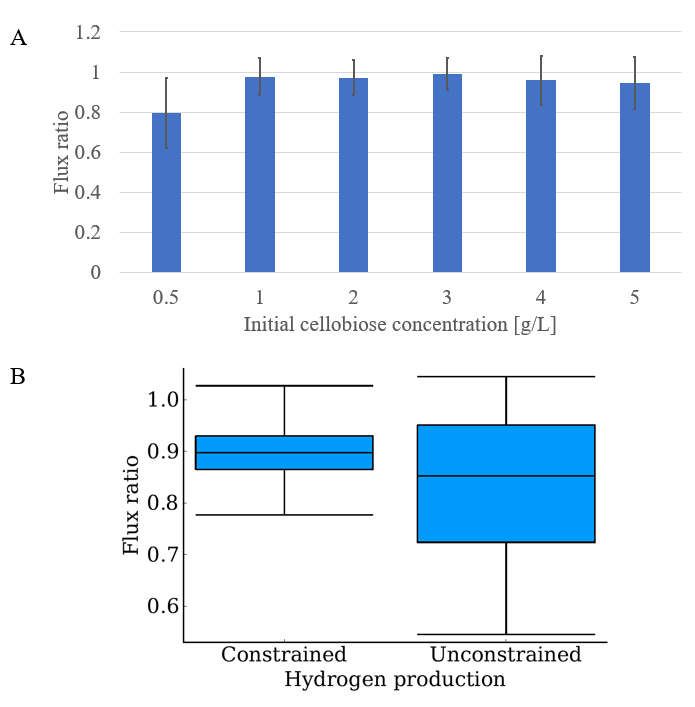

Supplement: FIG S6 [file msystems.00002-21-sf006.tif]
