# Supplementary material for: Experimentally Validated Reconstruction and Analysis of a Genome-Scale Metabolic Model of an Anaerobic Neocallimastigomycota Fungus
Source: mSystems. 2021 Feb 16;6(1):e00002-21. doi: 10.1128/mSystems.00002-21 (PMC8561657; doi:10.1128/mSystems.00002-21)
Supplement: TABLE S2 [file msystems.00002-21-st002.docx]

| Confidence score | Description | Number of reactions that satisfy criterion |
| --- | --- | --- |
| 0 | Gap (includes exchange reactions and transporters) | 306 |
| 1 | Inferred from presence in other gut fungi | 35 |
| 2 | Sequence homology evidence | 19 |
| 3 | Sequence homology and gene expression evidence | 686 |
