# Supplementary material for: Experimentally Validated Reconstruction and Analysis of a Genome-Scale Metabolic Model of an Anaerobic Neocallimastigomycota Fungus
Source: mSystems. 2021 Feb 16;6(1):e00002-21. doi: 10.1128/mSystems.00002-21 (PMC8561657; doi:10.1128/mSystems.00002-21)
Supplement: TABLE S3 [file msystems.00002-21-st003.docx]

| Hydrogenase type in model | Mean H_2_ flux [mmol/g_DW_/h] | St. D. H_2_ flux [mmol/g_DW_/h] |
| --- | --- | --- |
| Unconstrained ferredoxin | 0.58 | 0.31 |
| Unconstrained bifurcating | 3.48 | 0.19 |
| Experimentally measured H_2_ flux | 0.10 | 0.06 |
