# Supplementary material for: Experimentally Validated Reconstruction and Analysis of a Genome-Scale Metabolic Model of an Anaerobic Neocallimastigomycota Fungus
Source: mSystems. 2021 Feb 16;6(1):e00002-21. doi: 10.1128/mSystems.00002-21 (PMC8561657; doi:10.1128/mSystems.00002-21)
Supplement: TABLE S4 [file msystems.00002-21-st004.docx]

| Metabolite flux (produced) | Lower bound [mmol/g_DW_/h] | Upper bound [mmol/g_DW_/h] |
| --- | --- | --- |
| Formate | 0.88 | 2.65 |
| Acetate | 0.70 | 1.49 |
| Ethanol | 0.73 | 1.78 |
| Lactate | 0 | 1.05 |
| H_2_ | 0 | 1.49 |
| Succinate | 0 | 0.49 |
